# Supplementary figures and images for: Perceptual elements in Penn & Teller’s “Cups and Balls” magic trick
Source: PeerJ. 2013 Feb 12;1:e19. doi: 10.7717/peerj.19 (PMC3628988; doi:10.7717/peerj.19)

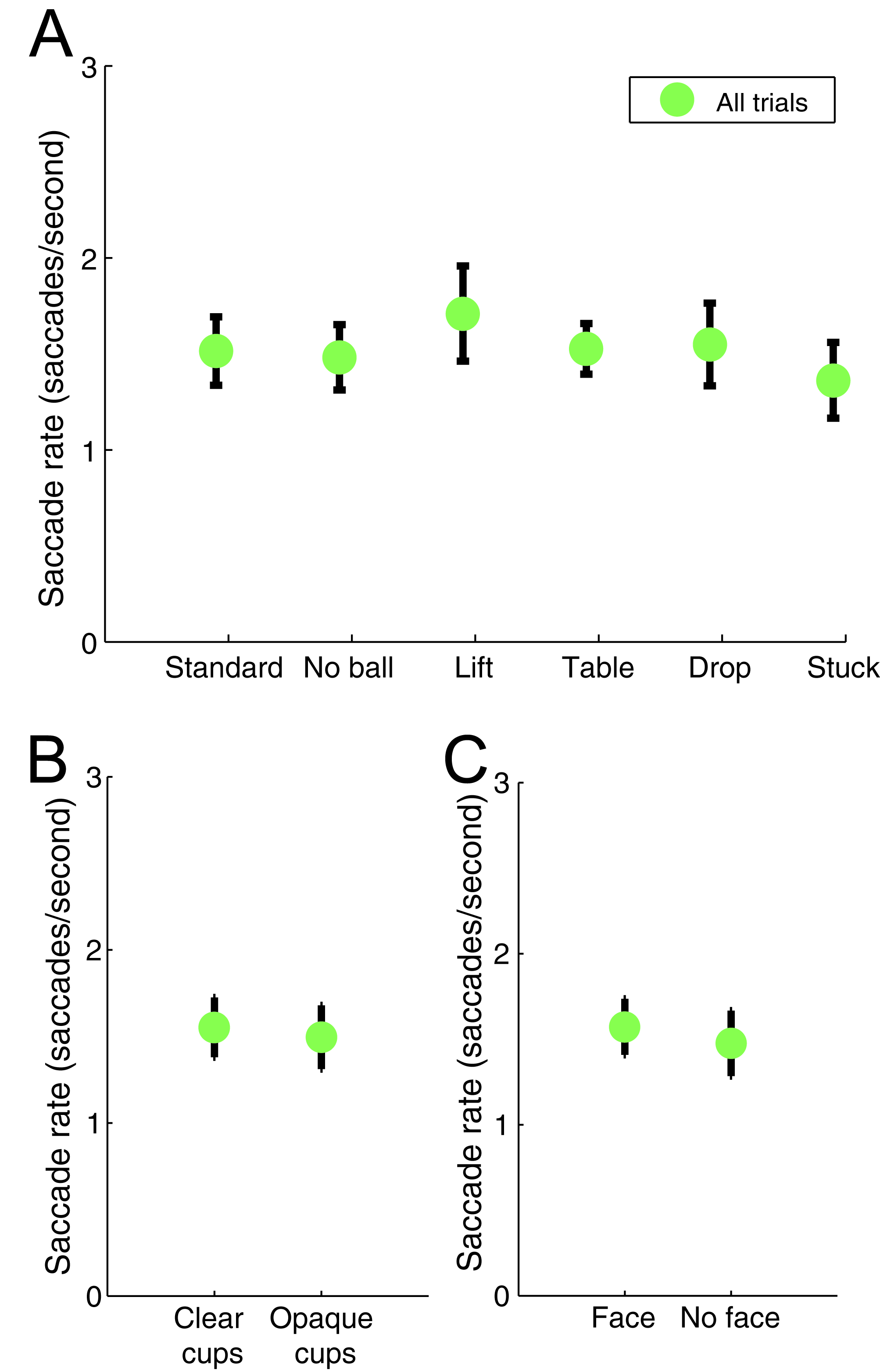

Supplement: Supplemental Figure S1 — a) Saccade rates across the different routines. There is no appreciable difference between routines. b) Saccade rate was similar for the “Clear Cups” and “Opaque Cups” conditions. c) Saccade rate was similar for the “Face” and “No face” conditions. Error bars indicate standard error from the mean across subjects. [file peerj-01-19-s001.jpg]

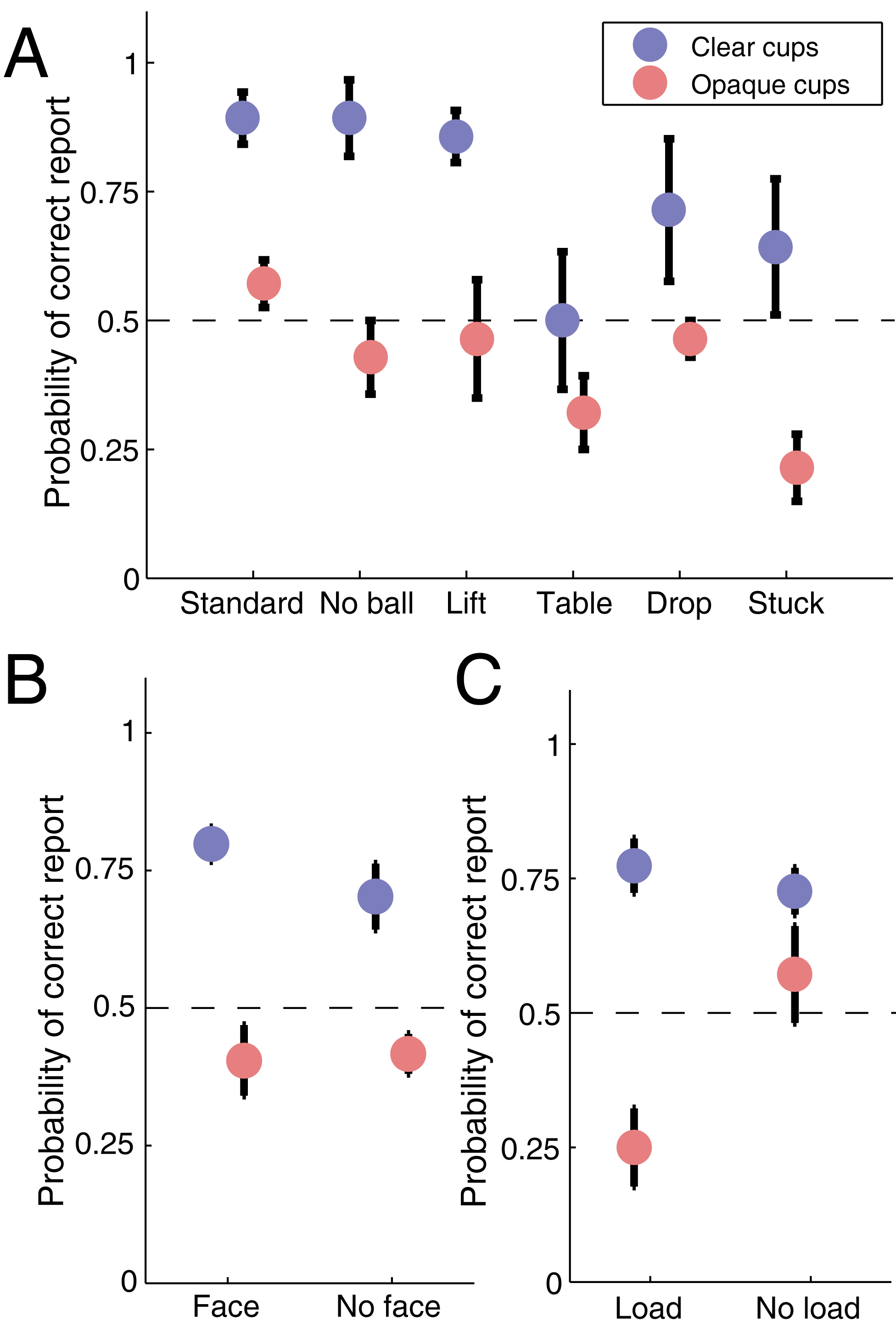

Supplement: Supplemental Figure S2 — a) Subjects accuracy in reporting the load of the third cup across the different routines, for the conditions with clear and opaque cups, for the first trial of each of the 48 conditions tested. Performance is uniform across the different routines for the opaque cups, and worse than in the conditions with clear cups (logistic regression, p < 10−7). When the cups are clear, and the loading or no loading of the cup is therefore visible, performance is worse for the “Table” and “Stuck” routines (logistic regression, p < 0.05). b) Performance was similar regardless of the face being visible or not. c) Performance was better for the “No load” condition with opaque cups (logistic regression, p < 0.001). Dashed lines show the expected chance performance level. Error bars indicate the standard error from the mean across subjects. [file peerj-01-19-s002.jpg]
